# Two Factors in Face Recognition: Whether You Know the Person’s Face and Whether You Share the Person’s Race

**DOI:** 10.1177/03010066211014016

**Published:** 2021-05-13

**Authors:** Xingchen Zhou, A. M. Burton, Rob Jenkins

**Affiliations:** University of York, UK

**Keywords:** face perception, memory, cognition, social cognition, familiarity, other-race effect

## Abstract

One of the best-known phenomena in face recognition is the other-race effect, the observation that own-race faces are better remembered than other-race faces. However, previous studies have not put the magnitude of other-race effect in the context of other influences on face recognition. Here, we compared the effects of (a) a race manipulation (own-race/other-race face) and (b) a familiarity manipulation (familiar/unfamiliar face) in a 2 × 2 factorial design. We found that the familiarity effect was several times larger than the race effect in all performance measures. However, participants expected race to have a larger effect on others than it actually did. Face recognition accuracy depends much more on whether you know the person’s face than whether you share the same race.

What makes a face hard to recognise? There is a common understanding among scientists, policy makers, and the general public that other-race faces are harder to recognise than own-race faces—a phenomenon known as the other-race effect (ORE; Meissner & Brigham, 2001). However, the importance of another critical factor, the viewer’s familiarity with a face (Burton et al., 2011; Natu & O’Toole, 2011), has not had such broad impact (Table 1). From a performance standpoint, the relative attention these effects have received is surprising, as it seems to invert their relative potency. Effects of familiarity (i.e., prior exposure to the person’s face) on face recognition are generally large (Bruce, 1982; Burton et al., 1999), whereas effects of race on face recognition are generally small (e.g., Sangrigoli et al., 2005; Tanaka et al., 2004).

**Table 1.** Prominence of race and familiarity effects.

Search term	Google	Google Scholar	Google Scholar all in title
Race face recognition	136,000,000	2,670,000	197
Familiarity face recognition	26,400,000	488,000	67
Race face recognition*-familiarity*	98,600,000	2,460,000	196
Familiarity face recognition*-race*	6,820,000	326,000	66

*Note*. Google search results (number of hits) for race-related and familiarity-related face recognition searches in general and specific sources. Note that a minus sign in the search term excludes the word that follows. For all searches, race hits outnumber familiarity hits. Similar comparisons yield similar results. Searches conducted June 2020.

One possible explanation for this inversion relates to the social importance of racial equality. The ORE is observed around the world for all known race combinations (Wan et al., 2015). But cognition does not occur in a vacuum, and there are social considerations surrounding race that may not apply to familiarity (Roberts & Rizzo, 2020). These considerations could raise the salience of experimental findings that are modulated by race, perhaps increasing the expectation that such effects will be cognitively large.

The accuracy of metacognitive insight becomes important here. Estimates of cognitive performance often diverge from actual cognitive performance, including face perception (Zhou & Jenkins, 2020). However, previous studies have rarely examined ORE and face familiarity in the same experiment, and few have incorporated metacognitive measures. As a result, the relative magnitude of these effects has not been a natural comparison. This is perhaps a missed opportunity, as such comparisons are informative. Research in face recognition seeks an understanding of factors that affect performance. But understanding *whether* a factor affects performance should also be accompanied by an attempt to understand *how much* it affects performance, and for the same reasons. Worlds in which one factor has 10%, 100%, or 1000% more impact than the other are meaningfully different, in terms of both theoretical understanding and applied implications. Eyewitness testimony is a prime example of an applied context where understanding memory is critical (Wells et al., 2006; Lindsay et al., 2011; http://www.innocenceproject.org). It is well documented that eyewitness testimony can be inaccurate, and it is important to establish the sources of potential error. The volume of writing on contributing factors (Table 1) might lead to an underemphasis on familiarity, by comparison to the ORE.

In the current experiments, we manipulated *race* and *familiarity* simultaneously in the same Old/New recognition memory experiments (Bower & Karlin, 1974; O’Toole et al., 1994). To gauge metacognitive insight into the effects of these factors, we asked participants to estimate their own performance on this task (self-estimates) and to estimate the performance of other participants (peer estimates). Consistent with the established literature, we expected to find a clear ORE and a clear familiarity effect. Based on reports of each effect separately (e.g., Bruce, 1982; Burton et al., 1999; Sangrigoli et al., 2005; Tanaka et al., 2004), we also expected that familiarity would be a stronger determinant of face recognition memory than race. Our main interest was the magnitude of this disparity. Given the relative prominence of these effects (Table 1), we also tested whether participants would overestimate effects of race relative to familiarity.

## Experiment 1 (Same Image at Learning and Test)

In this experiment, we used a standard face recognition memory procedure. Participants were shown a set of faces in a learning phase, followed by a test phase in which they were asked which faces they remembered seeing earlier. We manipulated both the race (own-race, other-race) and familiarity (familiar, unfamiliar) of the faces. As well as measuring recognition memory performance, we also asked participants to estimate the accuracy of their own and others’ performance on this memory task.

Many face recognition experiments have presented the same photograph of a face in the learning phase and the test phase. Such experiments depart from applied face recognition in that they can be solved by picture recognition (i.e., memory for a specific image). As picture recognition is generally good (Standing, 1973), this approach can lead to overestimates of face recognition accuracy (Bruce, 1982). However, our concern here was to connect with early research on the ORE (e.g., Malpass & Kravitz, 1969) and to facilitate comparisons across as broad a spectrum of literature as possible (Meissner & Brigham, 2001). For these reasons, we began by examining ORE and familiarity effects using a same-image design.

### Method

#### Participants

Sixty-four UK students (32 Black, 32 White; 48 female, 16 male; mean age 26 years; age range 18–61 years) from the University of York took part in the experiment in exchange for a small payment or course credit. The experiments in this study were approved by the Ethics Committee at the University of York. All participants provided written informed consent.

#### Stimuli and Design

Full colour face photographs of 32 Black celebrities, 32 White celebrities, 32 Black non-celebrities, and 32 White non-celebrities were downloaded from online sources (128 faces in total). Each of these categories contained 16 females and 16 males. The names of the celebrities are listed in Appendix. For each celebrity, we sought a non-celebrity whose face matched the same basic description. Each image was cropped and resized to 570 pixels high × 380 pixels wide for onscreen presentation. Experiments were run using a 21.5-inch iMac with i5 processor. Stimulus presentation and data collection were controlled by PsychoPy2 (Peirce et al., 2019).

To compare effects of race and familiarity directly, we constructed an Old/New recognition test in which the within-subjects factors of *Race* (*own*, *other*) and *Familiarity* (*familiar*, *unfamiliar*) were manipulated in a fully counterbalanced 2 × 2 factorial design. The experiment consisted of two main phases—a learning phase and a test phase. In the learning phase, participants viewed a series of 64 faces (16 Black celebrities, 16 White celebrities, 16 Black non-celebrities, 16 White non-celebrities) presented one at a time in a random order. In the subsequent test phase, participants viewed a longer series of 128 faces (32 Black celebrities, 32 White celebrities, 32 Black non-celebrities, 32 White non-celebrities) presented one at a time in a random order. Half of the faces in the test phase were *Old* faces that participants had seen in the learning phase. The other half were *New* faces that had not been presented before. Two complementary versions of the learning phase were counterbalanced across participants so that, when pooling over the whole experiment, each face appeared as an *Old* face and a *New* face an equal number of times.

We defined recognition performance as the proportion of correct responses in the memory test. To gauge metacognitive insight into the effects of each factor, we also recorded *self-estimates* and *peer estimates*. Self-estimates comprised three metrics. *Prospective* self-estimates were captured trial-by-trial in the learning phase. For each face, participants estimated the probability that they would recognise that face in the subsequent memory test (percentage response). *Concurrent* self-estimates were captured trial-by-trial in the test phase. For each face, participants rated their confidence that their own answer was correct (percentage response). *Retrospective* self-estimates were captured at debrief. The 64 faces from the learning phase were presented in a single array, grouped according to the 2 × 2 factorial design (16 Black celebrities, 16 White celebrities, 16 Black non-celebrities, 16 White non-celebrities; cell positions counterbalanced). Participants were asked to reflect on the task as a whole and to circle the two groups of faces they thought they remembered best. Choosing two of four options yields six possible combinations: 1–2, 1–3, 1–4, 2–3, 2–4, and 3–4.

Peer estimates were also captured trial-by-trial in the test phase. For each face, participants estimated (a) how many participants of the *same race* as the onscreen face would answer correctly, and (b) how many participants of *different race* to the onscreen face would answer correctly (both estimates out of 30).

#### Procedure

The experiment began with the learning phase, in which participants made prospective self-estimates for each of the 64 faces using a percentile scale. Following the learning phase, participants completed a short filler task (number search) before proceeding to the test phase. For each of the 128 faces in the test phase, participants made four separate responses in a fixed order: (i) whether the face was *Old* or *New* (recognition response), (ii) their confidence that their own recognition response was correct (concurrent self-estimate), (iii) the number of *same-race* participants they thought would give the correct recognition response (peer estimate), and (iv) the number of *different-race* participants they thought would give the correct recognition response (peer estimate). After completing the test phase, participants viewed all 128 faces again, indicating whether or not they already knew each face before the experiment (participants did not have to know the person’s name to know the person’s face). These familiarity responses were used to define *familiar* and *unfamiliar* faces for each participant. The familiarity check was followed by the retrospective self-estimate. Finally, participants were asked to write down their own ethnic group (free response). These ethnicity responses were used to define own-race and other-race faces for each participant. The experimenter provided task instructions at the beginning of each task. No time limit was imposed for any of the tasks. Each display remained on screen until the participant’s response, which immediately initiated the next trial. The entire test session took approximately 50 minutes to complete.

### Results

#### Overview

**Figure 1.**
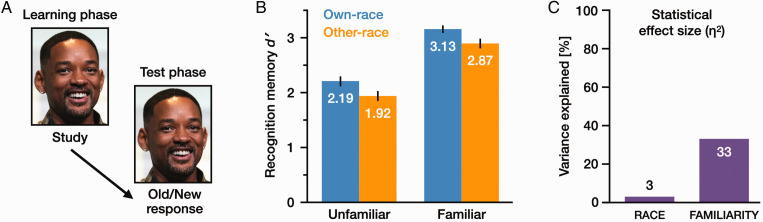
Memory performance in Experiment 1. (A) Each identity was represented by a single image, which was repeated in the learning phase and the test phase. (B) Mean recognition memory performance (*d′*) in each condition, and (C) statistical effect sizes (η^2^) for *Race* and *Familiarity*. Error bars show standard error (SE) [photo by Gage Skidmore CC BY-SA 3.0, https://commons.wikimedia.org/wiki/File:Will_Smith_by_Gage_Skidmore.jpg].

Data analyses for cognitive measures and metacognitive measures are reported in separate subsections later. For convenience, we present primary findings for memory performance in Figure 1.

**Figure 2.**
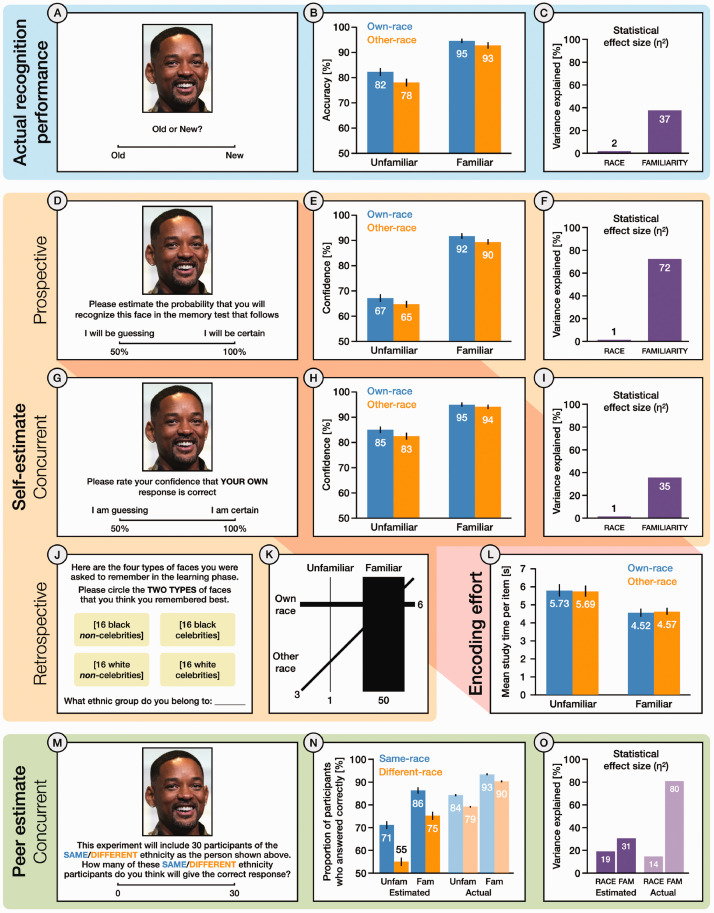
Summary methods and results for Experiment 1. Shaded regions show actual recognition performance (blue), self-estimates (yellow), and peer estimates (green). Example displays on the left, summary data on the right. Percentages rounded to nearest integer. Performance metrics show group means with standard error (SE). Statistical effect sizes show the proportion of total variance attributable to each effect (η^2^). (A) Actual recognition performance assessed via Old/New judgements to *Old* and *New* faces. (B) Percentage accuracy rates for each condition, and (C), statistical effect sizes for *Race* and *Familiarity* in the Old/New recognition memory test. (D) Prospective estimates of own recognition performance assessed via confidence judgements for each face at learning. (E) Percentage confidence ratings for each condition, and (F) statistical effect sizes for each factor in prospective estimates of own performance. (G) Concurrent estimates of own performance assessed via confidence judgements for each face at test. (H) Percentage confidence ratings for each condition, and (I) statistical effect sizes for each factor in concurrent estimates of own performance. (J) Retrospective estimates of own performance for each face type, captured after recognition test. (K) Combination plot showing the six possible ways to select two of the four face types in the retrospective self-estimate. Line thickness indicates frequency. (L) Mean study time per item for faces in each condition of the learning phase. (M) Concurrent estimates of peer performance for each test face. Participants provided separate estimates for peers who shared the depicted race (*Same*) and peers who did not (*Different*). (N) Estimated and actual peer performance for each condition, and (O) statistical effect sizes for each factor, shown separately for estimated and actual peer performance.

The statistical effect size (η^2^) for each factor indicates the proportion of variance explained by that factor (Figure 1C). Cohen’s (1988) ‘rules of thumb’ for interpreting effect sizes suggest an η^2^ value of .01 (1%) as a small effect, .06 (6%) as a medium effect, and .14 (14%) as a large effect. Following these guidelines, *Race* had a small effect (η^2^ = .03) and *Familiarity* had a large effect (η^2^ = .33). That is, the effect size was at least 10 times greater for *Familiarity* than for *Race*. Similar patterns were obtained for the two participant subgroups (see Supplemental Materials for subgroup analyses and raw data). Methods and results for all experimental measures are summarised in Figure 2.

#### Recognition Performance

We undertook three complementary analyses of recognition performance (Figure 2A), based on signal detection measures (Figure 1B and C), recognition accuracy (percentage of correct responses; Figure 2B and C), and the percentage of participants who answered each item correctly (Figure 2N and O). We also examined study time in the learning phase as a measure of encoding effort. Data from four participants (2 Black and 2 White), whose accuracy fell 3 *SD* below the group mean, were excluded from analysis. In all four analyses, effects of familiarity were much stronger than effects of race.

##### Signal Detection Analysis

A 2 × 2 repeated-measures analysis of variance (ANOVA) of *d'* values (Figure 1B) revealed a significant main effect of *Familiarity*, with higher *d'* values for *familiar* faces (*M *=* *3.00, standard error (*SE*)* *=* *0.07) than for *unfamiliar* faces (*M *=* *2.06, *SE *=* *0.09), *F*(1, 59) = 128.57, *p *<* *.001, η^2^ = .33. The main effect of *Race* was also significant, with higher *d'* for *own*-race faces (*M *=* *2.66, *SE *=* *0.07) than for *other*-race faces (*M *=* *2.40, *SE *=* *0.07), *F*(1, 59) = 26.55, *p *<* *.001, η^2^ = .03.

There was no significant interaction between these two factors, *F*(1, 59) = .01, *p *=* *.93, η^2^ < .01, suggesting that they are not strongly dependent on each other. A similar 2 × 2 ANOVA for Criterion *C* values also revealed a significant main effect of *Familiarity*, with a less strict criterion for *familiar* faces (*M *=* *.10, *SE *=* *0.03) than for *unfamiliar* faces (*M *=* *.53, *SE *=* *0.05), *F*(1, 59) = 70.62, *p *<* *.001, η^2^ = .26. Participants were more likely to classify *unfamiliar* faces as New and *familiar* faces as Old. There was no significant main effect of *Race*, *F*(1, 59) =.09, *p *=* *.* *77, η^2^ < .01, and no interaction between the two factors, *F*(1, 59) =.40, *p *=* *.* *53, η^2^ < .01. Descriptive statistics are shown in Table 2.

**Table 2.** Signal detection analysis for Experiment 1.

	Own-race face	Other-race face
*Hits*		
Familiar face	0.91 (0.01)	0.89 (0.01)
Unfamiliar face	0.68 (0.03)	0.63 (0.03)
*False alarms*		
Familiar face	0.05 (0.01)	0.09 (0.01)
Unfamiliar face	0.06 (0.01)	0.09 (0.01)
*d'*		
Familiar face	3.13 (0.07)	2.87 (0.09)
Unfamiliar face	2.19 (0.09)	1.92 (0.09)
*C*		
Familiar face	0.10 (0.03)	0.09 (0.04)
Unfamiliar face	0.51 (0.05)	0.54 (0.06)

*Note*. Mean hit rates, false alarms, computed *d'* values, and Criterion *C* values in each condition. Standard errors are shown in parentheses next to the respective means.

##### Accuracy

A 2 × 2 repeated-measures ANOVA with the factors of *Race* (*own*, *other*) and *Familiarity* (*familiar*, *unfamiliar*) also revealed a significant main effect of *Familiarity*, with higher accuracy for *familiar* faces (*M *=* *93.92, *SE *=* *0.71) than for *unfamiliar* faces (*M *=* *80.35, *SE *=* *1.24), *F*(1, 59) = 131.04, *p *<* *.001, η^2^ = .37. The main effect of *Race* was also significant, with higher accuracy for *own*-race faces (*M *=* *88.64, *SE *=* *0.85) than for *other*-race faces (*M *=* *85.62, *SE *=* *0.95), *F*(1, 59) = 17.12, *p *<* *.001, η^2^ = .02. There was no significant interaction between these the two factors, *F*(1, 59) = 3.26, *p *=* *.08, η^2^ < .01 (Figure 2B and C).

##### Proportion of Participants Who Answered Correctly

All proportions are expressed as percentages. A 2 × 2 repeated-measures ANOVA revealed a significant main effect of *Familiarity*, with more participants giving correct responses to *familiar* faces (*M *=* *91.18, *SE *=* *0.12) than to *unfamiliar* faces (*M *=* *81.70, *SE *=* *0.17), *F*(1, 59) = 4976.61, *p *<* *.001, η^2^ = .80, and a significant main effect of *Race,* with more participants giving correct responses to *same*-race faces (*M *=* *88.46, *SE *=* *0.11) than to *different*-race faces (*M *=* *84.43, *SE *=* *0.15), *F*(1, 59) = 8249.72, *p *<* *.001, η^2^ = .14. The interaction between *Familiarity* and *Race* was also significant, *F*(1, 59) = 80.10, *p *<* *.001, η^2^ < .01, with a larger effect of *Race* for *unfamiliar* faces than for *familiar* faces. Analysis of simple main effects showed that the effect of *Familiarity* was significant for both *same*-race faces, *F*(1, 59) = 2677.35, *p *<* *.001, η_p_^2^ = .98, and *different*-race faces, *F*(1, 59) = 5098.37, *p *<* *.001, η_p_^2^ = .99. The simple main effect of *Race* was significant for both *familiar* faces, *F*(1, 59) = 854.87, *p *<* *.001, η_p_^2^ = .94, and *unfamiliar* faces, *F*(1, 59) = 5530.45, *p *<* *.001, η_p_^2^ = .99 (Figure 2N and O).

##### Study Time in Learning Phase

A 2 × 2 repeated-measures ANOVA showed a significant main effect of *Familiarity*, with shorter study times for *familiar* faces (*M *=* *4.51, *SE* = 0.20) than for *unfamiliar* faces (*M *=* *5.71, *SE* = 0.32), *F*(1, 59) = 26.25, *p *<* *.001, η^2^ = .07. There was no significant main effect of *Race*, *F*(1, 59) < .01, *p *=* *.99, η^2^ < .01, and no interaction between the two factors, *F*(1, 59) = .15, *p *=* *.70, η^2^ < .01 (Figure 2L).

#### Self-Estimates

We conducted three separate analyses of self-estimates, based on *prospective* estimates (trial-by-trial confidence ratings in the learning phase; Figure 2D), *concurrent* estimates (trial-by-trial confidence ratings in the test phase; Figure 2G), and *retrospective* estimates (overall assessments at debrief; Figure 2J). In all three analyses, effects of familiarity were much stronger than effects of race.

##### Prospective Self-Estimates

A 2 × 2 repeated-measures ANOVA with the factors of *Race* (*own*, *other*) and *Familiarity* (*familiar*, *unfamiliar*) revealed a significant main effect of *Familiarity*, with higher confidence for *familiar* faces (*M *=* *90.71, *SE* = 0.79) than for *unfamiliar* faces (*M *=* *65.97, *SE* = 1.10), *F*(1, 59) = 555.49, *p *<* *.001, η^2^ = .72. The main effect of *Race* was also significant, with higher confidence for *own*-race faces (*M *=* *79.54, *SE* = 0.87) than for *other*-race faces (*M *=* *77.15, *SE* = 0.80), *F*(1, 59) = 27.33, *p *<* *.001, η^2^ = .01. There was no significant interaction between the two factors, *F*(1, 59) =.06, *p *=* *.* *81, η^2^ < .01 (Figure 2E and F).

##### Concurrent Self-Estimates

A 2 × 2 repeated-measures ANOVA revealed a significant main effect of *Familiarity*, with higher confidence for *familiar* faces (*M *=* *94.58, *SE* = 0.63) than for *unfamiliar* faces (*M *=* *83.76, *SE* = 1.05), *F*(1, 59) = 139.14, *p *<* *.001, η^2^ = .35. There was also a significant main effect of *Race*, with higher confidence for *own*-race faces (*M *=* *90.04, *SE* = 0.78) than for *other*-race faces (*M *=* *88.30, *SE* = 0.79), *F*(1, 59) = 10.20, *p *<* *.01, η^2^ = .01. Again, there was no significant interaction between the two factors, *F*(1, 59) = 2.03, *p *=* *.* *16, η^2^ <.01 (Figure 2H and I).

##### Retrospective Self-Estimates

Participants overwhelmingly chose *familiar, own-race* with *familiar, other-race,* indicating insight into the dominant effect of familiarity on their own performance. In contrast, the combination of *familiar, own-race* with *unfamiliar, own-race* was rarely chosen. A chi-square test confirmed that the frequencies for the six possible combinations were significantly different, χ^2^ (5, *N* = 60) = 109.73, *p *<* *.001 (Figure 2K).

#### Peer Estimates

All proportions are expressed as percentages. A 2 × 2 repeated-measures ANOVA revealed a significant main effect of *Familiarity*, with higher estimates for *familiar* faces (*M *=* *80.65, *SE *=* *1.23) than for *unfamiliar* faces (*M *=* *62.97, *SE *=* *1.31), *F*(1, 59) = 253.24, *p *<* *.001, η^2^ = .30. There was also a significant main effect of *Race*, with higher estimates for *same-race* (*M *=* *78.80, *SE *=* *1.24) than for *different-race* (*M *=* *64.82, *SE *=* *1.45), *F*(1, 59) = 95.63, *p *<* *.001, η^2^ = .19. The interaction between *Familiarity* and *Race* was also a significant, *F*(1, 59) = 47.70, *p *<* *.001, η^2^ = .01. Analysis of simple main effects showed that the effect of *Familiarity* was significant for both *same-race* faces, *F*(1, 59) = 186.70, *p *<* *.001, η_p_^2^ = .76, and *different-race* faces, *F*(1, 59) = 257.47, *p *<* *.001, η_p_^2^ = .81. The simple main effect of *Race* was significant for both *familiar* faces, *F*(1, 59) = 69.34, *p *<* *.001, η_p_^2^ = .54, and *unfamiliar* faces, *F*(1, 59) = 107.00, *p *<* *.001, η_p_^2^ = .65 (Figure 2N and O). In all our analyses of actual performance and self-estimates, *Race* had a small effect compared with *Familiarity*. For peer estimates, the two effects were more similar in magnitude (less than twofold disparity in statistical effect size; Figure 2O).

## Experiment 2 (Different Images at Learning and Test)

Experiment 1 demonstrated ORE and familiarity effects in the context of a same-image memory task. In Experiment 2, we presented different photos of each face in the learning phase and test phase, eliminating image-specific memory and requiring person memory (Bruce, 1982). Given that the appearance of an individual’s face changes continually (Jenkins et al., 2011), different-image tests more closely capture the problem of face recognition as it occurs in social and applied settings. Our main question was whether the pattern observed in Experiment 1 would generalise across this change.

### Method

#### Participants

Sixty-two UK students (32 Black, 30 White; 44 female, 18 male; mean age 22 years; age range 18–33 years) from the University of York took part in the experiment in exchange for a small payment or course credit. All participants provided written informed consent. None had participated in Experiment 1.

#### Stimuli and Design

**Figure 3.**
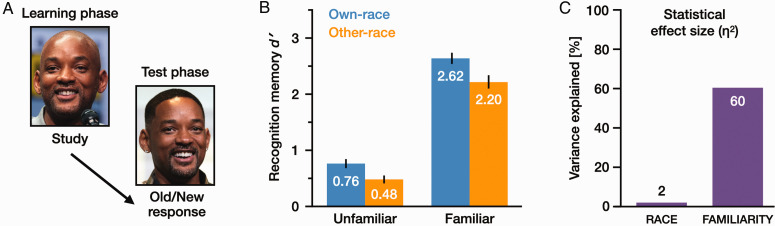
Memory performance in Experiment 2. (A) Each identity was represented by different images in the learning phase and the test phase. (B) Mean recognition memory performance (*d'*) in each condition, and (C) statistical effect sizes (η^2^) for *Race* and *Familiarity*. Error bars show standard error (SE) [photos by Gage Skidmore CC BY-SA 3.0, https://commons.wikimedia.org/wiki/File: Will_Smith_by_Gage_Skidmore.jpg; https://commons.wikimedia.org/wiki/File:Will_Smith_by_ Gage_Skidmore_2.jpg].

The stimuli and apparatus were the same as in Experiment 1, except that we now presented a different image of each face in the learning phase and the test phase (Figure 3A). The design and procedure were also the same as in Experiment 1, except that participants were now informed that different images of *Old* faces could be presented in the learning phase and the test phase.

### Results

#### Overview

Data analyses are presented using the same layout as for Experiment 1, with cognitive measures and metacognitive measures reported in separate subsections. Primary findings for memory performance are presented in Figure 3.

**Figure 4.**
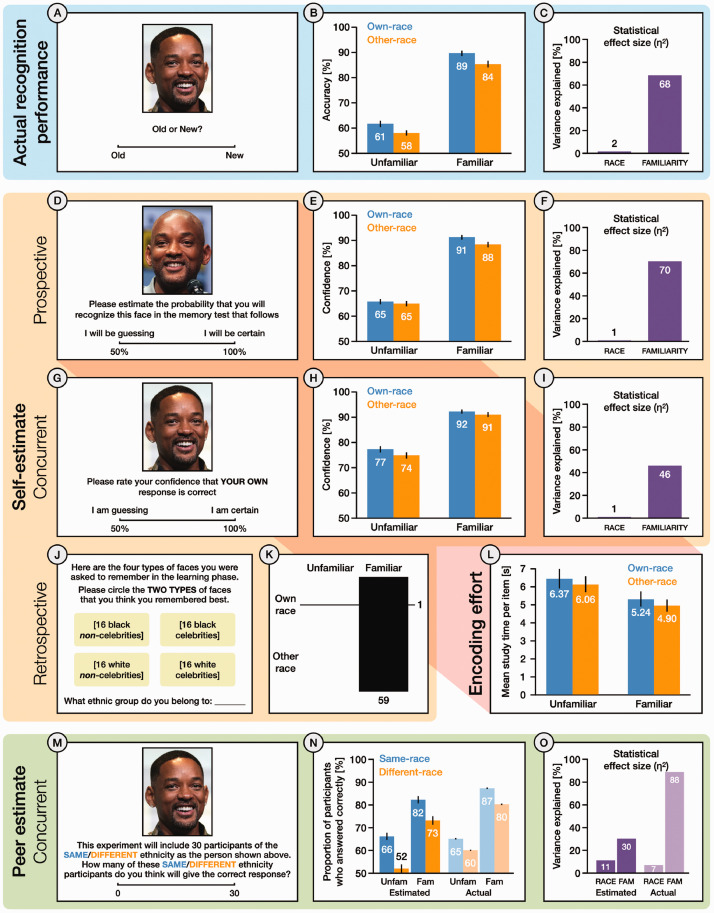
Summary methods and results for Experiment 2. (A) Actual recognition performance assessed via Old/New judgements to *Old* and *New* faces. (B) Percentage accuracy rates for each condition, and (C) statistical effect sizes for *Race* and *Familiarity* in the Old/New recognition memory test. (D) Prospective estimates of own recognition performance assessed via confidence judgements for each face at learning. (E) Percentage confidence ratings for each condition, and (F) statistical effect sizes for each factor in prospective estimates of own performance. (G) Concurrent estimates of own performance assessed via confidence judgements for each face at test. (H) Percentage confidence ratings for each condition, and (I) statistical effect sizes for each factor in concurrent estimates of own performance. (J) Retrospective estimates of own performance for each face type, captured after recognition test. (K) Combination plot showing the six possible ways to select two of the four face types in the retrospective self-estimate. Line thickness indicates frequency. (L) Mean study time per item for faces in each condition of the learning phase. (M) Concurrent estimates of peer performance for each test face. Participants provided separate estimates for peers who shared the depicted race (*Same*) and peers who did not (*Different*). (N) Estimated and actual peer performance for each condition, and (O) statistical effect sizes for each factor, shown separately for estimated and actual peer performance.

Statistical effect size (η^2^) for each factor is shown in Figure 3C. *Race* had a small effect (η^2^ = .02) and *Familiarity* had a large effect (η^2^ = .60). That is, the effect size was around 30 times greater for *Familiarity* than for *Race*. Similar patterns were obtained for the two participant subgroups (see Supplemental Materials for subgroup analyses and raw data). Methods and results for all experimental measures are summarised in Figure 4.

#### Recognition Performance

As with Experiment 1, we undertook three complementary analyses of recognition performance (Figure 4A), using signal detection measures (Figure 3B and C), recognition accuracy (percentage of correct responses; Figure 4B and C), and the percentage of participants who answered each item correctly (Figure 4N and O). We also examined study time in the learning phase as a measure of encoding effort. Data from two participants (both Black), whose accuracy fell 3 *SD* below the group mean, were excluded from analysis. Once again, effects of familiarity were much stronger than effects of race in all four analyses.

##### Signal Detection Analysis

A 2 × 2 repeated-measures ANOVA of *d′* values (Figure 3B) revealed a significant main effect of *Familiarity*, with higher *d′* values for *familiar* faces (*M *=* *2.41, *SE* = 0.10) than for *unfamiliar* faces (*M *=* *.62, *SE* = 0.07), *F*(1, 59) = 343.08, *p *<* *.001, η^2^ = .60. The main effect of *Race* was also significant, with higher *d'* for *own*-race faces (*M *=* *1.69, *SE* = 0.07) than for *other*-race faces (*M *=* *1.34, *SE* = 0.07), *F*(1, 59) = 35.51, *p *<* *.001, η^2^ = .02. There was no significant interaction between these two factors, *F*(1, 59) = 1.29, *p *=* *.26, η^2^ < .01, that is, no evidence that one factor is strongly dependent on the other. A similar 2 × 2 ANOVA for Criterion *C* values also revealed a significant main effect of *Familiarity*, with a less strict criterion for *familiar* faces (*M *=* *.08, *SE* = 0.04) than for *unfamiliar* faces (*M *=* *.65, *SE* = 0.06), *F*(1, 59) = 110.88, *p *<* *.001, η^2^ = .31. Participants were more likely to classify *unfamiliar* faces as New and *familiar* faces as Old. There was no significant main effect of *Race*, *F*(1, 59) = 2.15, *p *=* *.15, η^2^ < .01, and no interaction between the two factors, *F*(1, 59) = 2.67, *p *=* *.11, η^2^ < .01. Descriptive statistics are shown in Table 3.

**Table 3.** Signal detection analysis for Experiment 2.

	Own-race face	Other-race face
*Hits*		
Familiar face	0.87 (0.01)	0.81 (0.02)
Unfamiliar face	0.40 (0.03)	0.38 (0.02)
*False alarms*		
Familiar face	0.10 (0.01)	0.16 (0.02)
Unfamiliar face	0.18 (0.02)	0.23 (0.02)
*d'*		
Familiar face	2.62 (0.10)	2.20 (0.12)
Unfamiliar face	0.76 (0.08)	0.48 (0.07)
*C*		
Familiar face	0.08 (0.04)	0.08 (0.05)
Unfamiliar face	0.70 (0.07)	0.59 (0.06)

*Note*. Mean hit rates, false alarms, computed *d'* values, and Criterion *C* values in each condition. Standard errors are shown in parentheses next to the respective means.

##### Accuracy

A 2 × 2 repeated-measures ANOVA with the factors of *Race* (*own*, *other*) and *Familiarity* (*familiar*, *unfamiliar*) also revealed a significant main effect of *Familiarity*, with higher accuracy for *familiar* faces (*M *=* *86.84, *SE *=* *1.12) than for *unfamiliar* faces (*M *=* *59.61, *SE *=* *0.98), *F*(1, 59) = 489.16, *p *<* *.001, η^2^ = .68. The main effect of *Race* was also significant, with higher accuracy for *own*-race faces (*M *=* *75.33, *SE* = 0.93) than for *other*-race faces (*M *=* *71.12, *SE* = 0.95), *F*(1, 59) = 29.28, *p *<* *.001, η^2^ = .02. There was no significant interaction between these the two factors, *F*(1, 59) = .54, *p *=* *.46, η^2^ < .01 (Figure 4B and C).

##### Proportion of Participants Who Answered Correctly

All proportions are expressed as percentages. A 2 × 2 repeated-measures ANOVA revealed a significant main effect of *Familiarity*, with more participants giving correct responses to *familiar* faces (*M *=* *83.46, *SE *=* *0.31) than to *unfamiliar* faces (*M *=* *62.21, *SE *=* *0.30), *F*(1, 59) = 3737.02, *p *<* *.001, η^2^ = .88, and a significant main effect of *Race,* with more participants giving correct responses to *same*-race faces (*M *=* *75.87, *SE *=* *0.25) than to *different*-race faces (*M *=* *69.80, *SE *=* *0.26), *F*(1, 59) = 22101.59, *p *<* *.001, η^2^ = .08. The interaction between *Familiarity* and *Race* was also significant, *F*(1, 59) = 96.61, *p *<* *.001, η^2^ < .01, reflecting a larger effect of *Race* for *unfamiliar* faces than for *familiar* faces. Analysis of simple main effects showed that the effect of *Familiarity* was significant for both *same*-race faces, *F*(1, 59) = 4216.74, *p *<* *.001, η_p_^2^ = .99, and *different*-race faces, *F*(1, 59) = 2783.93, *p *<* *.001, η_p_^2^ = .98. The simple main effect of *Race* was significant for both *familiar* faces, *F*(1, 59) = 2952.43, *p *<* *.001, η_p_^2^ = .98, and *unfamiliar* faces, *F*(1, 59) = 2811.24, *p *<* *.001, η_p_^2^ = .98 (Figure 4N and O).

##### Study Time in Learning Phase

A 2 × 2 repeated-measures ANOVA showed a significant main effect of *Familiarity*, with shorter study times for *familiar* faces (*M *=* *5.07, *SE* = 0.35) than for *unfamiliar* faces (*M *=* *6.22, *SE* = 0.48), *F*(1, 59) = 15.29, *p *<* *.001, η^2^ = .03, and a significant main effect of *Race*, with shorter study times for *other*-race faces (*M *=* *5.48, *SE* = 0.37) than for *own*-race faces (*M *=* *5.81, *SE* = 0.44), *F*(1, 59) = 4.58, *p *<* *.05, η^2^ < .01. There was no interaction between the two factors, *F*(1, 59) = .01, *p *=* *.92, η^2^ < .01 (Figure 4L).

#### Self-Estimates

As for Experiment 1, we conducted three separate analyses of self-estimates, based on *prospective* estimates (trial-by-trial confidence ratings in the learning phase; Figure 4D), *concurrent* estimates (trial-by-trial confidence ratings in the test phase; Figure 4G), and *retrospective* estimates (overall assessments at debrief; Figure 4J). In all three analyses, effects of familiarity were much stronger than effects of race.

##### Prospective Self-Estimates

A 2 × 2 repeated-measures ANOVA with the factors of *Race* (*own*, *other*) and *Familiarity* (*familiar*, *unfamiliar*) revealed a significant main effect of *Familiarity*, with higher confidence for *familiar* faces (*M *=* *89.33, *SE* = 0.97) than for *unfamiliar* faces (*M *=* *65.07, *SE* = 0.99), *F*(1, 59) = 445.71, *p *<* *.001, η^2^ = .70. The main effect of *Race* was also significant, with higher confidence for *own*-race faces (*M *=* *78.12, *SE* = 0.90) than for *other*-race faces (*M *=* *76.28, *SE* = 0.86), *F*(1, 59) = 15.48, *p *<* *.001, η^2^ < .01. The interaction between *Familiarity* and *Race* was also significant, *F*(1, 59) = 4.16, *p *<* *.05, η^2^ < .01, with a larger effect of *Race* for *familiar* faces than for *unfamiliar* faces.

Analysis of simple main effects showed that the effect of *Familiarity* was significant for both *own*-race faces, *F*(1, 59) = 420.76, *p *<* *.001, η_p_^2^ = .88, and *other*-race faces, *F*(1, 59) = 340.31, *p *<* *.001, η_p_^2^ = .85. The simple main effect of *Race* was significant for *familiar* faces, *F*(1, 59) = 16.62, *p *<* *.001, η_p_^2^ = .22, but not for *unfamiliar* faces, *F*(1, 59) = 1.73, *p *=* *.19, η_p_^2^ = .03 (Figure 4E and F).

##### Concurrent Self-Estimates

A 2 × 2 repeated-measures ANOVA revealed a significant main effect of *Familiarity*, with higher confidence for *familiar* faces (*M *=* *91.15, *SE* = 0.84) than for *unfamiliar* faces (*M *=* *75.68, *SE* = 1.21), *F*(1, 59) = 204.58, *p *<* *.001, η^2^ = .46. There was also a significant main effect of *Race*, with higher confidence for *own*-race faces (*M *=* *84.32, *SE* = 0.88) than for *other*-race faces (*M *=* *82.51, *SE* = 0.96), *F*(1, 59) = 17.16, *p *<* *.001, η^2^ = .01. There was no significant interaction between the two factors, *F*(1, 59) = 1.59, *p *=* *.* *21, η^2^ < .01 (Figure 4H and I).

##### Retrospective Self-Estimates

Participants overwhelmingly chose *familiar, own-race* with *familiar, other-race,* indicating insight into the dominant effect of familiarity on their own performance. In contrast, the combination of *familiar, own-race* with *unfamiliar, own-race* was rarely chosen. A chi-square test confirmed that the frequencies for the six possible combinations were significantly different, χ^2^ (1, *N* = 60) = 56.07, *p *<* *.001 (Figure 4K).

#### Peer Estimates

All proportions are expressed as percentages. A 2 × 2 repeated-measures ANOVA revealed a significant main effect of *Familiarity*, with higher estimates for *familiar* faces (*M *=* *77.76, *SE *=* *1.63) than for *unfamiliar* faces (*M *=* *58.69, *SE *=* *1.58), *F*(1, 59) = 227.38, *p *<* *.001, η^2^ = .30. There was also a significant main effect of *Race*, with higher estimates for *same-race* (*M *=* *73.91, *SE *=* *1.50) than for *different-race* (*M *=* *62.53, *SE *=* *1.68), *F*(1, 59) = 88.99, *p *<* *.001, η^2^ = .11. The interaction between *Familiarity* and *Race* was also a significant, *F*(1, 59) = 34.57, *p *<* *.001, η^2^ = .01, with a larger effect of *Race* for *unfamiliar* faces than for *familiar* faces. Analysis of simple main effects showed that the effect of *Familiarity* was significant for both *same-race* faces, *F*(1, 59) = 225.91, *p *<* *.001, η_p_^2^ = .79, and *different-race* faces, *F*(1, 59) = 194.37, *p *<* *.001, η_p_^2^ = .77. The simple main effect of *Race* was significant for both *familiar* faces, *F*(1, 59) = 62.70, *p *<* *.001, η_p_^2^ = .52, and *unfamiliar* faces, *F*(1, 59) = 92.84, *p *<* *.001, η_p_^2^ = .61 (Figure 4N and O). In all our analyses of actual performance and self-estimates, *Race* had a small effect compared with *Familiarity*. For peer estimates, the two effects were again more similar in magnitude (less than twofold disparity in statistical effect size; Figure 4O).

## General Discussion

In two experiments, we examined the impact of a race manipulation (own-race/other-race face) and a familiarity manipulation (familiar/unfamiliar face) on face recognition memory. In Experiment 1 (same image at learning and test), recognition performance was affected by race as expected. However, the influence of familiarity was much stronger. Comparison of effect sizes revealed at least a 10-fold dominance of familiarity over race in determining recognition performance. This disparity was present in signal detection measures and percentage accuracy and was not explained by differences in encoding effort during the learning phase (Wan et al., 2015). In Experiment 2 (different images at learning and test), a similar pattern emerged, demonstrating that the results in Experiment 1 were not due to image repetition. For this more realistic memory task, the dominance of familiarity over race was at least 30-fold.

Self-estimates showed that participants were aware that familiarity affected their own performance more than race. In both experiments, trial-by-trial confidence ratings from the learning phase and the test phase tracked actual performance, as did retrospective estimates at debrief.

Peer estimates revealed a very different pattern. Consistent with previous work, participants systematically underestimated other people’s performance (Alicke et al., 1995). More important, they overestimated ORE, giving similar weight to race effects as to familiarity effects (less than twofold disparity).

That the ORE is a statistically small effect is not news (Meissner & Brigham, 2001; Rossion & Michel, 2011). But other cognitively small effects in the literature (e.g., own-age bias, Rhodes & Anastasi, 2012; own-gender bias, Herlitz & Lovén, 2013) have not acquired the same burden of expectation as ORE. Despite widespread belief to the contrary, matching or mismatching racial group membership does not have a large effect on face recognition memory. In comparison, familiarity has a much larger effect on face recognition memory—not by a few percent, but by at least an order of magnitude in terms of statistical effect size.

Such comparisons are helpful if we are to understand errors of mistaken identity and calibrate expectations appropriately. Consider identification judgements from two eyewitnesses. Witness A is familiar with the individual concerned but does not share the same race. Witness B shares the same race but is not familiar with the individual. Our findings suggest that the identification judgement by Witness A is more likely to be reliable.

As well as bringing applied questions into focus, the relative strength of psychological effects also informs theoretical progress and model development. For example, order of magnitude differences are often explicitly coded in qualitative reasoning systems (Ali et al., 2003; Raiman, 1990), and appropriate weighting of inputs is essential to the operation of neural networks. The issue of modelling psychological variables is timely, given the recent focus on racial bias in face recognition algorithms (Cavazos et al., 2021). While familiarity is a stronger predictor than race in human face recognition, it does not have a clear analogue in machine systems (Jenkins & Burton, 2008).

The central claim in this article is that familiarity matters more than race in face recognition memory. Every part of that claim contributes to its intended meaning. First, the claim concerns only relative importance. We do not argue that familiarity is the most important factor or that race is the least important factor. Second, the claim concerns only face recognition memory. The statistical effect size of the ORE has no bearing on the social importance of the ORE. Interestingly, metacognitive measures could shed some light on this key distinction. Participants in our experiments expected race to have a larger effect on others than it actually did. If the social importance of the ORE reflects *beliefs* about its impact on performance, as opposed to its *actual* impact on performance, this could help to reconcile its small effect size with its high profile.

For the avoidance of doubt, we are not seeking to minimise OREs. The magnitude of the ORE we report here is absolutely consistent with previous observations. Instead, we are seeking to maximise the familiarity effect. The direct comparison here underscores the transformative effect of familiarity on cognition. If you want to become good at recognising a person’s face, become familiar with that person’s face.

## Supplemental Material

sj-pdf-1-pec-10.1177_03010066211014016 - Supplemental material for Two Factors in Face Recognition: Whether You Know the Person’s Face and Whether You Share the Person’s Race Click here for additional data file. Supplemental material, sj-pdf-1-pec-10.1177_03010066211014016 for Two Factors in Face Recognition: Whether You Know the Person’s Face and Whether You Share the Person’s Race by Xingchen Zhou, A. M. Burton and Rob Jenkins in Perception

## Appendix: List of Celebrities

### Black Celebrities

*Counterbalancing Version A*. Alicia Keys, Beyoncé, Cardi B, Jada Pinkett Smith, Mel B, Oprah Winfrey, Serena Williams, Zendaya, Barack Obama, Drake, Dwayne Johnson, Idris Elba, Kendrick Lamar, Morgan Freeman, The Weeknd, will.i.am.

*Counterbalancing Version B*. Ariana Grande, Jennifer Hudson, Kelly Rowland, Mariah Carey, Michelle Obama, Nicki Minaj, Rihanna, Whitney Houston, Eddie Murphy, Jay-Z, Kanye West, Kevin Hart, Michael B. Jordan, Samuel L. Jackson, Travis Scott, Will Smith.

### White Celebrities

*Counterbalancing Version A.* Angelina Jolie, Ellen DeGeneres, Emma Watson, Hillary Clinton, Julia Roberts, Katy Perry, Kristen Stewart, Miley Cyrus, Benedict Cumberbatch, Chris Hemsworth, David Beckham, Harry Styles, Justin Bieber, Leonardo DiCaprio, Rowan Atkinson, Rupert Grint.

*Counterbalancing Version B.* Theresa May, Emma Stone, Scarlett Johansson, Selena Gomez, Anne Hathaway, Adele, Lady Gaga, Taylor Swift, Brad Pitt, Daniel Radcliffe, Donald Trump, Ed Sheeran, Gary Barlow, Martin Freeman, Robert Downey Jr., Ryan Reynolds.
