# Supplementary material for: Two Factors in Face Recognition: Whether You Know the Person’s Face and Whether You Share the Person’s Race
Source: Perception. 2021 May 13;50(6):524–39. doi: 10.1177/03010066211014016 (PMC8371284; doi:10.1177/03010066211014016)
Supplement: sj-pdf-1-pec-10.1177_03010066211014016 - Supplemental material for Two Factors in Face Recognition: Whether You Know the Person’s Face and Whether You Share the Person’s Race [file sj-pdf-1-pec-10.1177_03010066211014016.pdf]

Supplementary Materials for *Two factors in face recognition: Whether you know the person's face and whether you share the person's race*, by Zhou, Burton, and Jenkins

Separate analysis of Black and White subgroups in each experiment shows similar ORE and familiarity effects in each subgroup.

Experiment 1

Black participants

Signal detection analysis. A 2 × 2 repeated-measures ANOVA of *d'* values revealed a significant main effect of *Familiarity*, with higher *d'* values for *familiar* faces (M = 2.81, SE = .12) than for *unfamiliar* faces (M = 1.97, SE = .11) [F (1, 29) = 65.53, p < .001, η^2 = .28]. The main effect of *Race* was also significant, with higher *d'* for *own*-race faces (M = 2.49, SE = .09) than for *other*-race faces (M = 2.28, SE = .12) [F (1, 29) = 7.93, p < .01, η^2 = .02]. There was no significant interaction between these the two factors [F (1, 29) = .21, p = .65, η^2 < .01] (see Figure S1A, Figure S1B).

Accuracy. A 2 × 2 repeated-measures ANOVA with the factors of *Race* (*own*, *other*) and *Familiarity* (*familiar*, *unfamiliar*) also revealed a significant main effect of *Familiarity*, with higher accuracy for *familiar* faces (M = 92.02, SE = 1.23) than for *unfamiliar* faces (M = 78.74, SE = 1.78) [F (1, 29) = 63.75, p < .001, η^2 = .33]. The main effect of *Race* was also significant, with higher accuracy for *own*-race faces (M = 86.93, SE = 1.24) than for *other*-race faces (M = 83.82, SE = 1.57) [F (1, 29) = 6.64, p < .05, η^2 = .02]. There was no significant interaction between these the two factors [F (1, 29) = .69, p = .41, η^2 < .01] (see Figure S1C, Figure S1D).

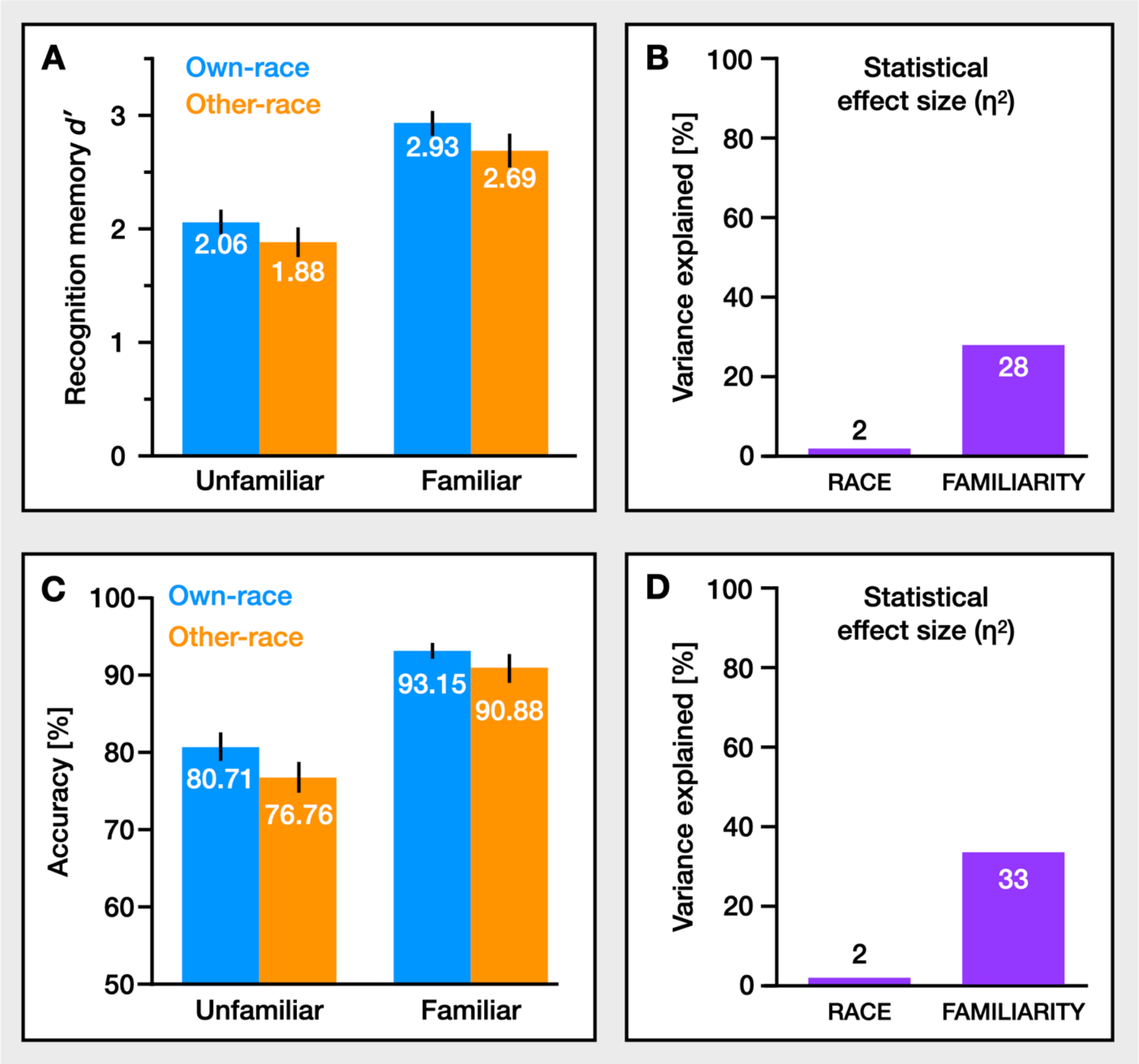

Figure S1. Results for black participants in Experiment 1. (A) Mean recognition memory performance (*d′*) in each condition, and (B) its statistical effect sizes (η^2) for *Race* and *Familiarity* of black participants in Experiment 1. (C) Percentage accuracy rates for each condition, and (D) its statistical effect sizes (η^2) for *Race* and *Familiarity* of black participants in Experiment 1.

White participants

Signal detection analysis. A 2 × 2 repeated-measures ANOVA of *d′* values revealed a significant main effect of *Familiarity*, with higher *d′* values for *familiar* faces (M = 3.19, SE = .07) than for *unfamiliar* faces (M = 2.15, SE = .13) [F (1, 29) = 65.28, p < .001, η^2 = .41]. The main effect of *Race* was also significant, with higher *d′* for *own*-race faces (M = 2.83, SE

= .09) than for *other*-race faces (*M* = 2.51, *SE* = .08) [*F* (1, 29) = 20.49, *p* < .001, η^2 = .04]. There was no significant interaction between these the two factors [*F* (1, 29) = .34, *p* = .56, η^2 < .01] (see Figure S2A, Figure S2B).

Accuracy. A 2 × 2 repeated-measures ANOVA with the factors of *Race* (*own*, *other*) and *Familiarity* (*familiar*, *unfamiliar*) also revealed a significant main effect of *Familiarity*, with higher accuracy for *familiar* faces (*M* = 95.82, *SE* = .57) than for *unfamiliar* faces (*M* = 81.96, *SE* = 1.72) [*F* (1, 29) = 65.21, *p* < .001, η^2 = .44]. The main effect of *Race* was also significant, with higher accuracy for *own*-race faces (*M* = 90.36, *SE* = 1.09) than for *other*-race faces (*M* = 87.42, *SE* = .98) [*F* (1, 29) = 12.04, *p* < .01, η^2 = .02]. There was no significant interaction between these the two factors [*F* (1, 29) = 3.39, *p* = .08, η^2 < .01] (see Figure S2C, Figure S2D).

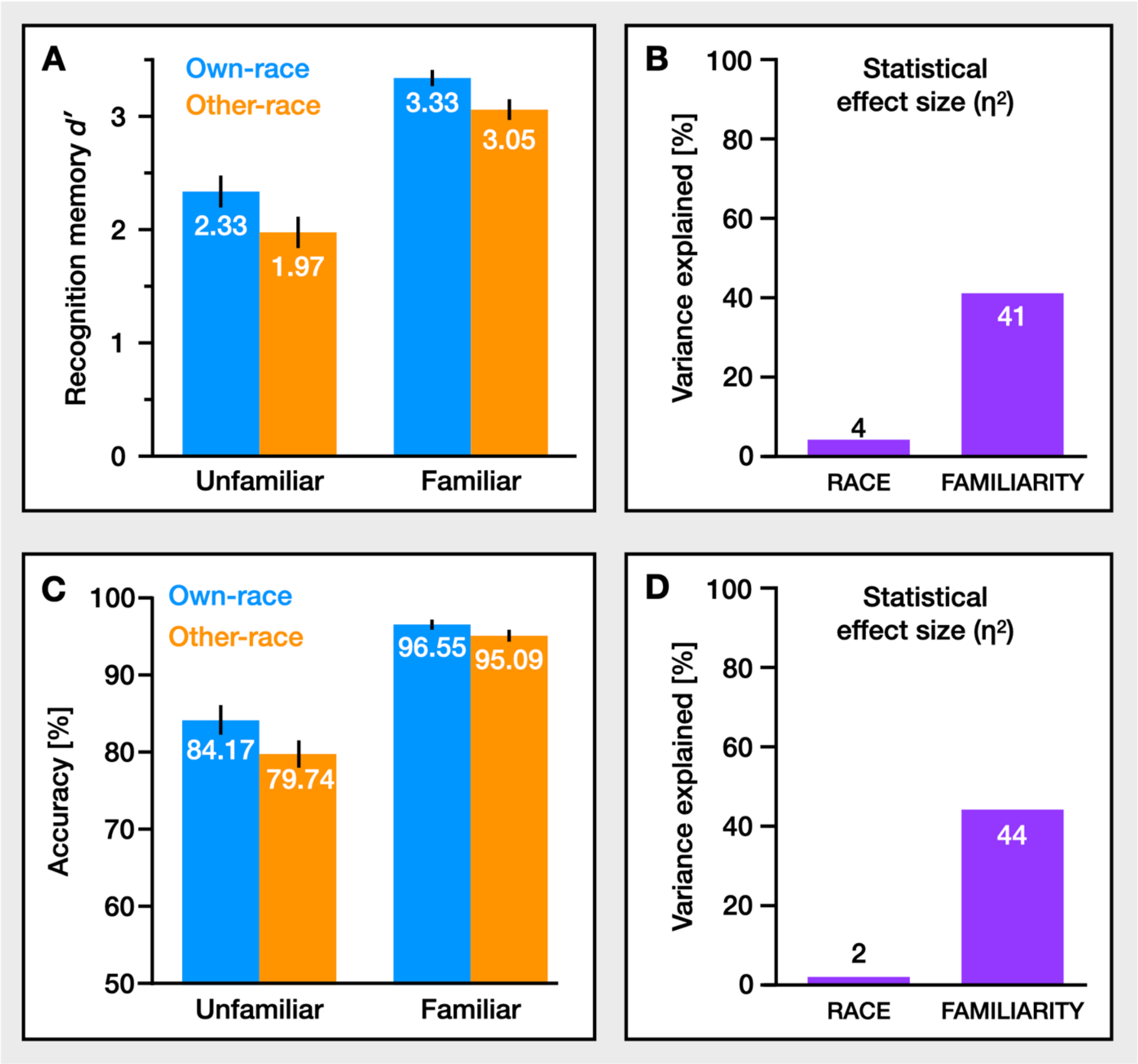

Figure S2. Results for white participants in Experiment 1. (A) Mean recognition memory performance (*d′*) in each condition, and (B) its statistical effect sizes (η^2) for *Race* and *Familiarity* of white participants in Experiment 1. (C) Percentage accuracy rates for each condition, and (D) its statistical effect sizes (η^2) for *Race* and *Familiarity* of white participants in Experiment 1.

Experiment 2

Black participants

Signal detection analysis. A 2 × 2 repeated-measures ANOVA of *d'* values revealed a significant main effect of *Familiarity*, with higher *d'* values for *familiar* faces (M = 2.21, SE = .14) than for *unfamiliar* faces (M = .64, SE = .09) [F (1, 29) = 145.45, p < .001, η^2 = .53]. The main effect of *Race* was also significant, with higher *d'* for *own*-race faces (M = 1.58, SE = .11) than for *other*-race faces (M = 1.26, SE = .10) [F (1, 29) = 11.25, p < .01, η^2 = .02]. There was no significant interaction between these the two factors [F (1, 29) = .19, p = .67, η^2 < .01] (see Figure S3A, Figure S3B).

Accuracy. A 2 × 2 repeated-measures ANOVA with the factors of *Race* (*own*, *other*) and *Familiarity* (*familiar*, *unfamiliar*) also revealed a significant main effect of *Familiarity*, with higher accuracy for *familiar* faces (M = 83.87, SE = 1.70) than for *unfamiliar* faces (M = 60.37, SE = 1.46) [F (1, 29) = 198.51, p < .001, η^2 = .58]. The main effect of *Race* was also significant, with higher accuracy for *own*-race faces (M = 74.45, SE = 1.55) than for *other*-race faces (M = 69.79, SE = 1.41) [F (1, 29) = 13.82, p < .01, η^2 = .02]. There was no significant interaction between these the two factors [F (1, 29) = .01, p = .94, η^2 < .01] (see Figure S3C, Figure S3D).

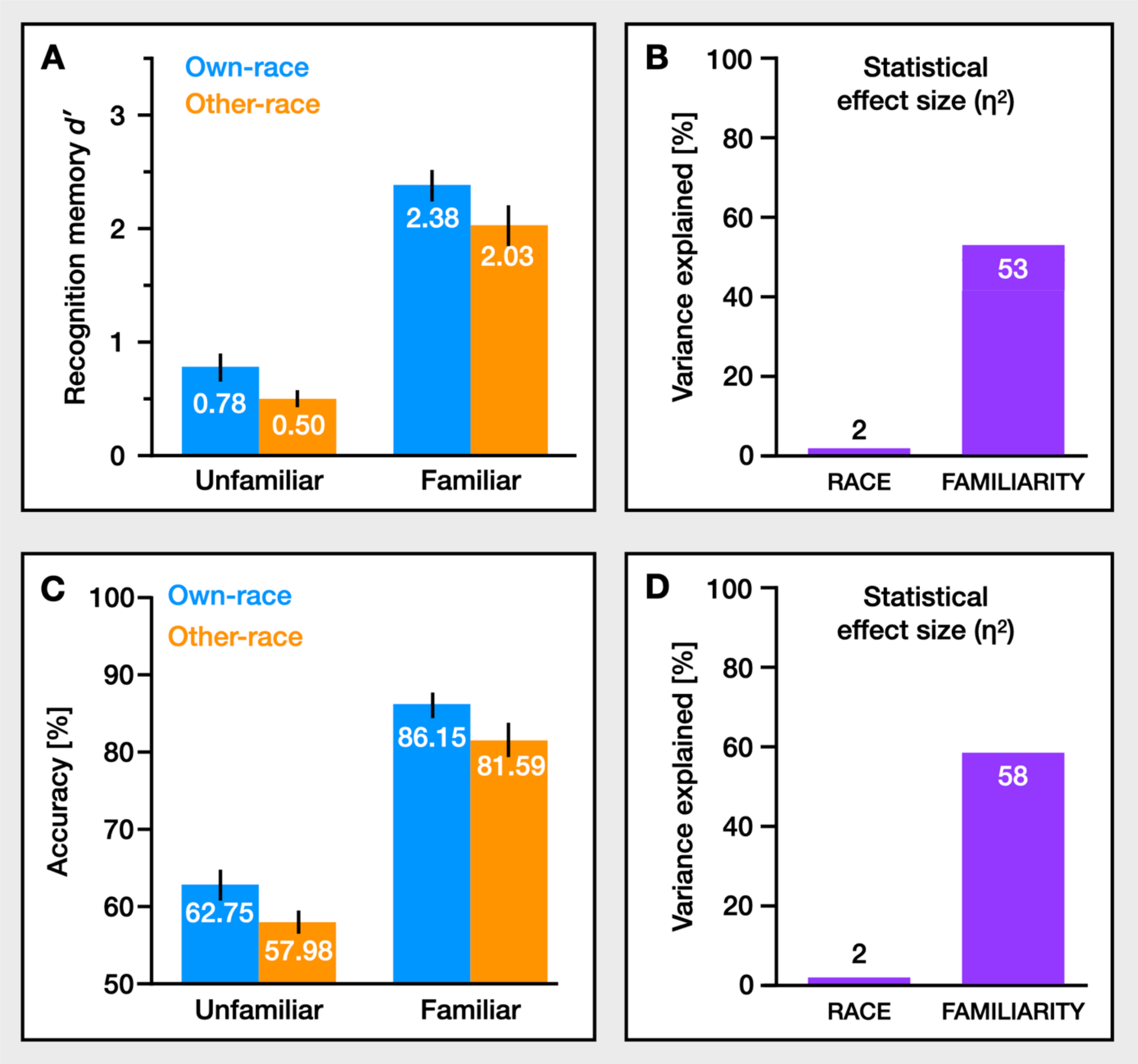

Figure S3. Results for black participants in Experiment 2. (A) Mean recognition memory performance (*d′*) in each condition, and (B) its statistical effect sizes (η^2) for *Race* and *Familiarity* of black participants in Experiment 2. (C) Percentage accuracy rates for each condition, and (D) its statistical effect sizes (η^2) for *Race* and *Familiarity* of black participants in Experiment 2.

White participants

Signal detection analysis. A 2 × 2 repeated-measures ANOVA of *d′* values revealed a significant main effect of *Familiarity*, with higher *d′* values for *familiar* faces (*M* = 2.62, *SE*

= .12) than for *unfamiliar* faces (M = .61, SE = .10) [F (1, 29) = 229.42, p < .001, η^2 = .68]. The main effect of *Race* was also significant, with higher d' for *own*-race faces (M = 1.80, SE = .09) than for *other*-race faces (M = 1.42, SE = .09) [F (1, 29) = 29.33, p < .001, η^2 = .02]. There was no significant interaction between these the two factors [F (1, 29) = 1.28, p = .27, η^2 < .01] (see Figure S4A, Figure S4B).

Accuracy. A 2 × 2 repeated-measures ANOVA with the factors of *Race* (*own*, *other*) and *Familiarity* (*familiar*, *unfamiliar*) also revealed a significant main effect of *Familiarity*, with higher accuracy for *familiar* faces (M = 89.80, SE = 1.27) than for *unfamiliar* faces (M = 58.86, SE = 1.33) [F (1, 29) = 394.36, p < .001, η^2 = .79]. The main effect of *Race* was also significant, with higher accuracy for *own*-race faces (M = 76.22, SE = 1.02) than for *other*-race faces (M = 72.45, SE = 1.25) [F (1, 29) = 16.12, p < .001, η^2 = .01]. There was no significant interaction between these the two factors [F (1, 29) = 1.93, p = .18, η^2 < .01] (see Figure S4C, Figure S4D).

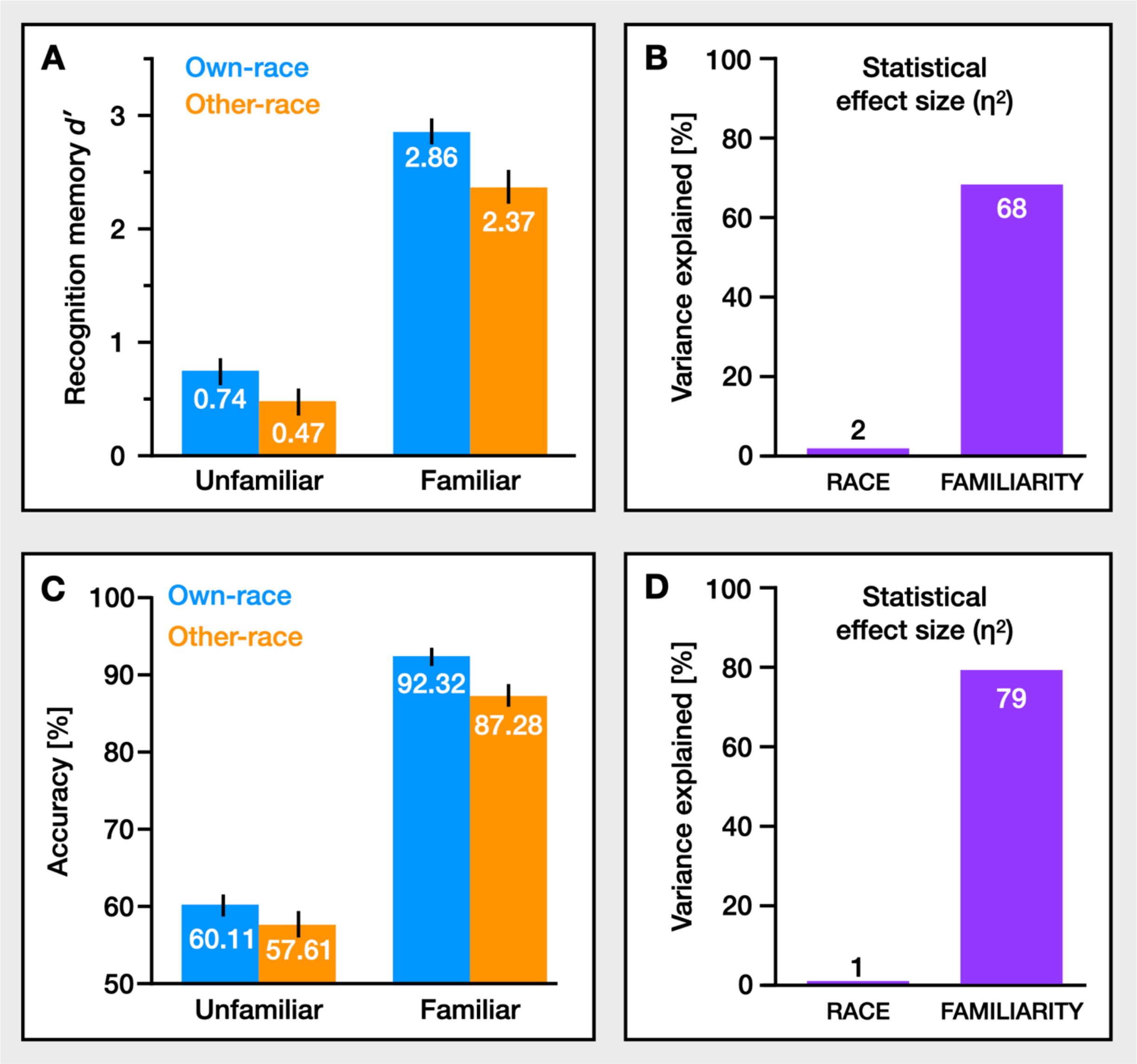

Figure S4. Results for white participants in Experiment 2. (A) Mean recognition memory performance (*d′*) in each condition, and (B) its statistical effect sizes (η^2) for *Race* and *Familiarity* of white participants in Experiment 2. (C) Percentage accuracy rates for each condition, and (D) its statistical effect sizes (η^2) for *Race* and *Familiarity* of white participants in Experiment 2.
